# RGO-Coated Polyurethane Foam/Segmented Polyurethane Composites as Solid–Solid Phase Change Thermal Interface Material

**DOI:** 10.3390/polym12123004

**Published:** 2020-12-16

**Authors:** Cong Zhang, Zhe Shi, An Li, Yang-Fei Zhang

**Affiliations:** Department of Materials Science and Engineering, College of Engineering, Peking University, Beijing 100871, China; 1601214778@pku.edu.cn (C.Z.); 1801214052@pku.edu.cn (Z.S.); lian1993@pku.edu.cn (A.L.)

**Keywords:** reduced graphene oxide, segmented polyurethane, composites, solid–solid phase change, thermal interface material, thermal conductivity, interfacial wettability

## Abstract

Thermal interface material (TIM) is crucial for heat transfer from a heat source to a heat sink. A high-performance thermal interface material with solid–solid phase change properties was prepared to improve both thermal conductivity and interfacial wettability by using reduced graphene oxide (rGO)-coated polyurethane (PU) foam as a filler, and segmented polyurethane (SPU) as a matrix. The rGO-coated foam (rGOF) was fabricated by a self-assembling method and the SPU was synthesized by an in situ polymerization method. The pure SPU and rGOF/SPU composite exhibited obvious solid–solid phase change properties with proper phase change temperature, high latent heat, good wettability, and no leakage. It was found that the SPU had better heat transfer performance than the PU without phase change properties in a practical application as a TIM, while the thermal conductivity of the rGOF/SPU composite was 63% higher than that of the pure SPU at an ultra-low rGO content of 0.8 wt.%, showing great potential for thermal management.

## 1. Introduction

Thermal management is crucial for the electronic industries, and strongly affects the performance, reliability, and lifetime of devices with high integration and power density [1]. Because of the surface roughness of electronic chips and heat sinks, the real contact area between them is less than 10% [2], seriously affecting the thermal contact resistance, causing heat concentration on the chip surface and thermal induced failures. A thermal interface material (TIM), mainly composed of polymer matrixes and thermal conductive fillers such as metals, ceramics, carbon materials, and hybrid fillers, can be applied between the heat source and the heat sink to fill the voids and grooves caused by imperfections of mating surfaces, thus minimizing the thermal contact resistance [3,4]. With its excellent mechanical, electrical, and thermal properties, graphene has become an ideal reinforcement material of composites [5,6,7]. With the advantages of high thermal conductivity and a lightweight nature, graphene composites have attracted great attention in thermal management applications [8,9,10]. In recent years, three-dimensional (3D) interconnected structures of graphene, forming large numbers of conduction paths at low content [11,12], have been developed rapidly and used for thermal conduction in many TIMs. Many methods have been developed to prepare 3D interconnected structures, including self-assembly, freeze drying, and chemical vapor deposition [13,14,15,16,17,18].

The performance of TIMs depends on both thermal conductivity and contact resistance [4]. Because their good wettability and low modulus of elasticity can increase the contact area and reduce the contact resistance, thermal greases are widely used, alongside traditional phase change materials (PCMs), which can become liquid and fill gaps and voids at high temperatures [19,20,21]. However, thermal greases are limited by the problems of pump-out and dry-out [22], while solid–liquid PCMs have the disadvantages of leakage and extra encapsulations [23]. The use of solid–solid PCMs is an excellent solution to these problems [24,25,26,27,28]. Segmented polyurethane (SPU) with solid–solid phase change properties, a new and rapidly developing material, is a kind of block copolymer, composed of polyethylene glycol (PEG) with high molecular weight as the soft segment, 4,4′-diphenylmethane diisocyanate (MDI) as the hard segment, and 1,4-butanediol (BDO) as the chain extender [29]. When the temperature is higher than the phase change temperature, the SPU transforms into an amorphous solid phase and becomes soft, which leads to good interfacial wettability. Furthermore, the high latent heat and proper phase change temperature makes SPU attractive for many applications, including thermal energy storage, thermal conduction, thermal interface, and thermal insulation. [30,31,32]

In this work, a reduced graphene oxide (rGO)-coated foam (rGOF)-filled SPU composite was prepared as a high-performance thermal interface material with solid–solid phase change properties. The rGOF was fabricated by a self-assembling method and its microstructure and chemical properties were investigated by a scanning electron microscope (SEM) and Raman spectra. The SPU as a matrix was synthesized by an in situ polymerization method. The properties of the SPU and the rGOF/SPU composite were investigated by differential scanning calorimetry (DSC), polarizing optical microscopy (POM), and a thermal conductance meter. A self-development in situ test system was used to study their performances in a practical application as a TIM. This work has provided a method to greatly improve the heat transfer performance of thermal interface material and thermal conductive polymer composites for thermal management.

## 2. Materials and Methods

### 2.1. Materials

Commercial polyurethane (PU) sponge with a density of 18 mg·cm^−3^ and a pore diameter of 1 mm was used in this work. Flake graphite (325 mesh, 99%) was purchased from Qingdao Laixi graphite Co. Ltd., Qingdao, China. Polyethylene glycol (PEG; Mn = 6000) and 4,4′-diphenylmethane diisocyanate (MDI) were purchased from Shanghai Aladdin Chemical Reagent Co. Ltd., Shanghai, China. The 1,4-butanediol (BDO) was purchased from Fisher Scientific Worldwide (Shanghai) Co., Ltd, Shanghai, China. Other reagents of analytical grade were purchased from Sinopharm Chemical Reagent Co. Ltd, Shanghai, China.

### 2.2. Preparation of the rGOF/SPU Composite

**Figure 1:**
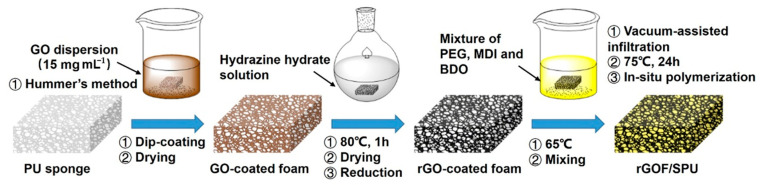
Schematic illustration of the synthetic route to preparing the reduced graphene oxide (rGO)-coated foam (rGOF)/segmented polyurethane (rGOF/SPU) composite. PU, polyurethane.

Figure 1 shows a schematic illustration of the synthetic route to preparing the rGOF/SPU composite. The graphene oxide (GO) was synthesized by a modified Hummer’s method. Concentrated H_2_SO_4_ (46 mL) was added to the mixture of flake graphite (2 g) and NaNO_3_ (3 g) in an ice bath. KMnO_4_ (6 g) was added slowly with the temperature of reaction system below 5 °C. Then, the mixture was heated to 35 °C for 2 h with stirring. Deionized water (150 mL) was added to the flask and the temperature was maintained at 98 °C for 30 min. The mixture was then diluted with deionized water (500 mL), and 30% H_2_O_2_ was added gradually until the solution turned yellow. The product was washed by HCl (1 mol·L^−1^) to remove metallic ions, and by deionized water several times until the pH reached 7. The GO was dried by lyophilization.

Flexible PU foam was then immersed into hydrazine hydrate for several minutes until the color turned from yellow to white, and was then washed with deionized water. The dye was removed during decolorizing to facilitate the binding between the GO and PU foams. After drying in a vacuum oven, the foam was immersed in a GO colloidal solution of a relatively high concentration (15 mg·mL^−1^) and turned dark brown. A GO-coated foam (GOF) was obtained after drying in open conditions. The reduction of GO was reacted in hydrazine hydrate solution at 80 °C for 1 h. The foam turned black after reaction and was washed in deionized water several times. The obtained rGOF was placed in a vacuum oven until completely dry.

Finally, the rGOF/SPU composite was obtained by an in situ polymerization method. The PEG was degassed and dried under a vacuum (−100 kPa) at 100 °C for 2 h. The MDI (1.79 g) and the chain extender BDO (added by drops) were introduced into melted PEG (7.68 g) and stirred. The rGOF was infiltrated into the mixture using a vacuum-assisted method. The temperature was raised to 75 °C for 24 h to promote curing. An rGOF/SPU composite with good structural integrity and excellent properties was obtained. According to the weights of the pure foam, rGOF, and rGOF/SPU composite, the rGO content in the composite was calculated as an ultra-low value of 0.8 wt.%.

### 2.3. Material Characterizations

The microstructures of the foam and composite were observed by a scanning electron microscope (SEM) (S4800, Hitachi Co., Tokyo, Japan) with an accelerating voltage and a current set as 5 kV and 10 μA, respectively. The Raman spectra were measured by a Micro Raman imaging spectrometer (DXRxi, ThermoFisher Co., Waltham, MA, USA) with a laser of 532 nm and power of 5 mW. The exposure time was set as 0.5 s, and the step number was 100. The observation of polarizing optical microscopy (POM) was performed using a transmitted polarization microscope (59XC-PC, Shanghai Optical Co. Ltd., Shanghai, China) equipped with a halogen lamp of 765 nm. The sample was placed on a coverslip and heated with a constant temperature heating plate (KER 3100-08S, Nanjing Kaier Co. Ltd., Nanjing, China). Differential scanning calorimetry (DSC) was performed on a differential scanning calorimeter (DSC 8000, PerkinElmer Co., Waltham, MA, USA) with a heating rate of 10 °C min^−1^ over a temperature range between room temperature and 100 °C. Thermal conductivity was measured by the heat flux method using a thermal conductance meter (DRL-III, Hunan Xiangyi Instruments Co. Ltd., Xiangtan, China) in accordance with ASTM D5470 standard test method. A pressure of 170 kPa was applied to enhance the thermal contact between the sample and the copper bars. The temperatures of the hot end and the cold end were 50 °C and 20 °C, respectively. Each test was repeated three times to obtain the average value.

## 3. Results and Discussion

**Figure 2:**
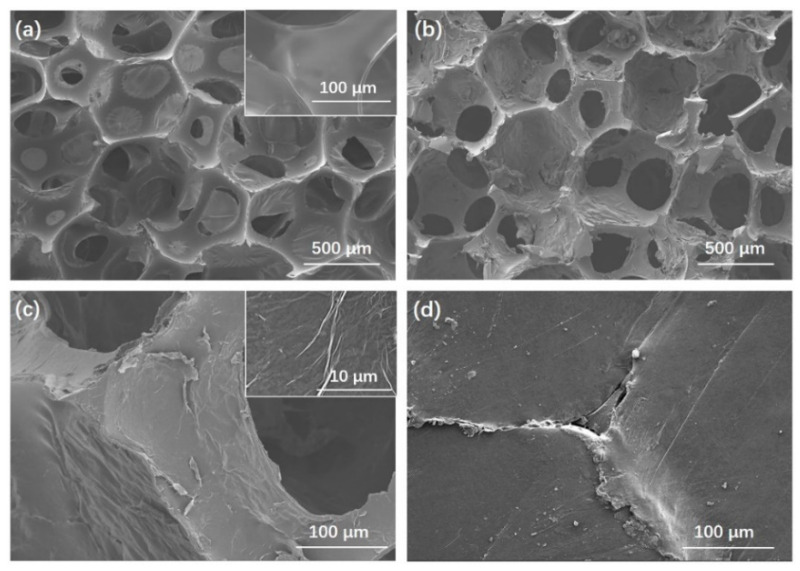
Scanning electron microscope (SEM) images of PU foam (**a**); rGOF (**b**,**c**) with different resolutions; the cutting edge of the rGOF/SPU composite (**d**).

The internal morphology of the PU foam, rGOF, and rGOF/SPU composite were observed by the SEM. The three-dimensional porous structure of the PU foam was clearly observed with a pore size of several hundred microns (Figure 2a). The smooth surface of the PU foam skeleton indicated good impurity and no coating, which is beneficial to the self-assembling of GO. After dip-coating and reduction, the skeleton surface became rough and wrinkled (Figure 2b,c), suggesting successful coating of rGO on the PU skeleton. The wrinkled structure was attributed to the huge specific surface area of graphene, and is consistent with other works [33]. As graphene is flexible and easy to curl, the rGO-coated PU foam inherited the porous structure and flexibility of PU foam. A three-dimensional interconnected structure with continuous rGO coated on the surface is advantageous to heat transfer by formation through thermal conduction paths at low content [6,34]. The rGOF observed at the cutting edge of the rGOF/SPU composite showed that the rGOF was completely embedded in the SPU matrix without obvious defect, which is beneficial for thermal and mechanical properties (Figure 2d).

**Figure 3:**
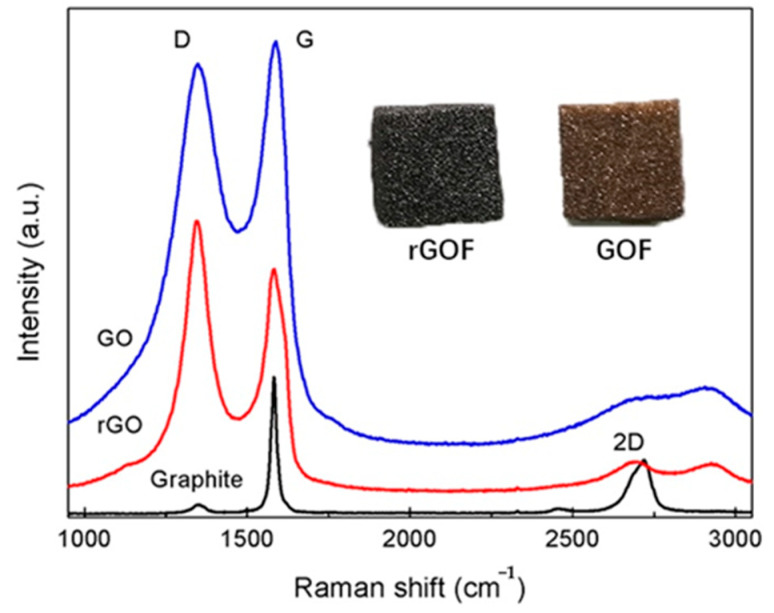
Raman spectra of natural graphite, GO and rGO and images of GOF and rGOF.

Raman spectra were used to investigate the reduction in GO. From the Raman spectra of the GO and the rGO, two main bands were found at approximately 1350 cm^−1^ and 1580 cm^−1^, corresponding to D band and G band, respectively [35] (Figure 3). The D band is usually regarded as a reflection of disorder and defects in a carbon structure, while the G band as a signature of the graphitic component [36]. Compared to the spectra of graphite, the intensity ratio of the D band to the G band (ID/IG) increased from 0.96 to 1.19 after chemical reduction using hydrazine hydrate. This was attributed to the smaller size of the rGO and some remaining functionalities, or to increasing defects after reduction [37]. The 2D band of the rGO of approximately 2700 cm^−1^ also suggested the restoration of sp^2^ carbon structure [38]. The color change from dark brown to black is also evidence of a reduction.

**Figure 4:**
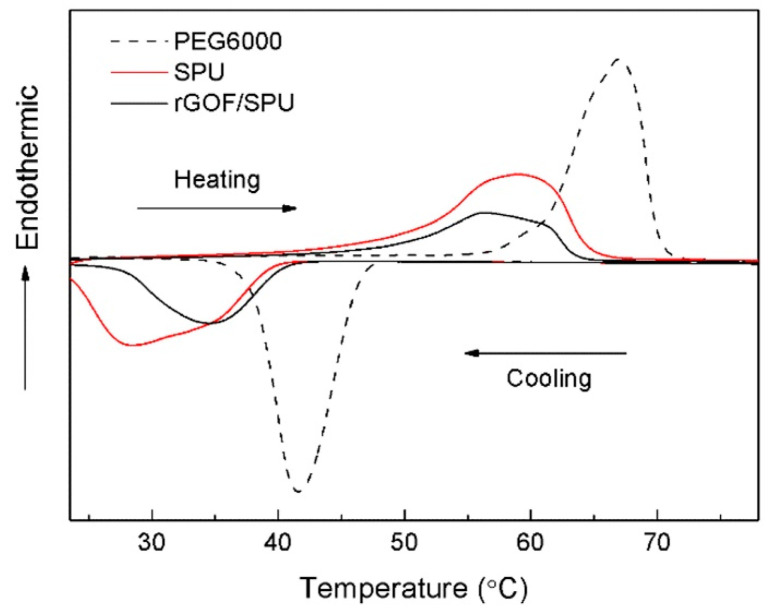
Differential scanning calorimeter (DSC) curves of PEG6000, SPU, and rGOF/SPU.

Figure 4 shows the DSC results of the pure PEG6000, SPU, and rGOF/SPU composite. The latent heat of the rGOF/SPU composite during the heating and cooling cycles was 61.12 J·g^−1^ and 58.54 J·g^−1^, respectively. Compared with the SPU, the phase change properties of the rGOF/SPU composite were not influenced significantly by adding rGOF. The melting peak of the rGOF/SPU composite occurred at 56.3 °C and was well matched to the working temperature range of electronic chips. As the temperature rose, solid–solid phase change occurred and lots of heat was absorbed as latent heat. The SPU, which is hard at room temperature, turned soft and easy to reshape in an amorphous solid state.

**Figure 5:**
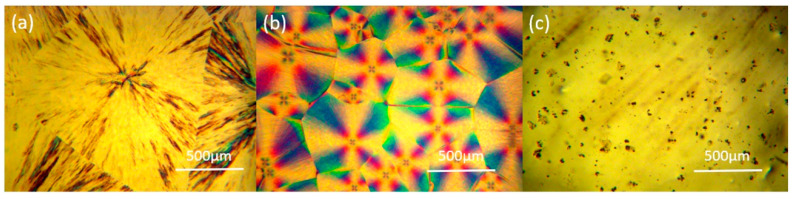
Polarizing optical microscopy (POM) micrographs of PEG6000 (**a**), SPU (**b**) at room temperature, and SPU at 60 °C (**c**).

The crystalline morphology of the pure PEG6000 and SPU at room temperature was observed by POM micrographs (Figure 5a,b). The obvious cross-extinction patterns in both micrographs indicated their spherulites crystal structures, consistent with other works [29,39]. The size of the spherulites of the SPU was several hundred microns, much smaller than that of the PEG. As a block copolymer, the crystallization of the PEG in the SPU was restricted by the hard segment, and the crystalline perfection was damaged. This phenomenon was also reflected by a smaller transition enthalpy of the SPU and the rGOF/SPU composite. When heated to 60 °C (above the phase change temperature), the spherulite structures of the SPU disappeared because the soft segments had become amorphous.

**Figure 6:**
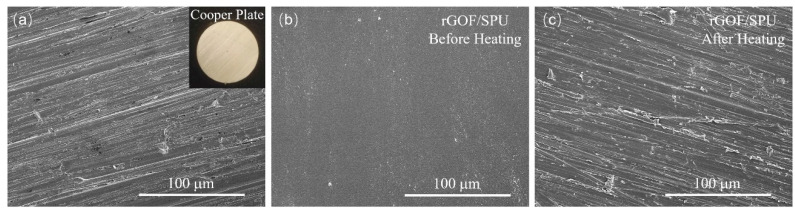
SEM images of the surfaces of the copper plate (**a**) and the rGOF/SPU composite before (**b**) and after (**c**) heating.

To visualize the interfacial wettability of the rGOF/SPU composite, the sample was heated between two copper plates at 80 °C, simulating a TIM being between a heat source and a heat sink. The surfaces of the copper plates and the rGOF/SPU before and after heating were observed by the SEM. Figure 6 shows that the surface of the rGOF/SPU before heating is smooth, and then becomes matching with the surface morphology of copper plate after heating, due to the solid–solid phase change of polyurethane. The soft segments of the SPU block copolymer became amorphous and filled the interface voids when heating was applied, showing good wetting characteristics and a low modulus of elasticity to help eliminate the thermal contact resistance [2]. 

The thermal conductivities measured by the heat flux method were 0.27 W·m^−1^·K^−1^ and 0.44 W·m^−1^·K^−1^ for the SPU and the rGOF/SPU composite, respectively. An increase in thermal conductivity by 63% was achieved at an ultra-low graphene content of 0.8 wt.%. This was because continuous coating of the rGO constructed an effective heat transfer network from surface to surface and provided many thermal conduction paths, which makes 3D interconnected structures of graphene an ideal filler in thermal conductive composites [40].

**Figure 7:**
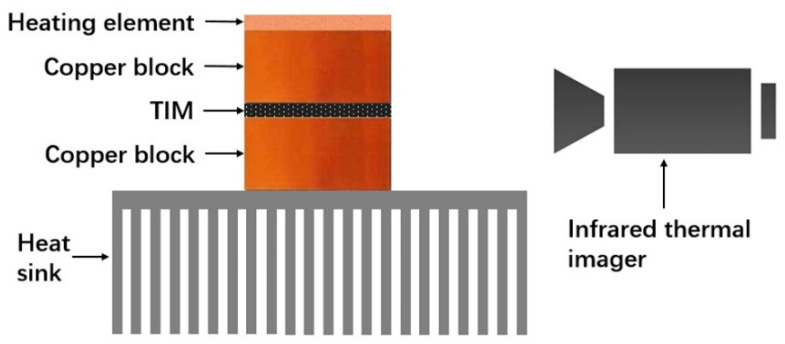
Schematic illustration of a self-development in situ test system. TIM, thermal interface material.

A self-development in situ test system based on infrared thermal imaging technology was used to measure the performance of the rGOF/SPU composite in working conditions as a TIM. Figure 7 shows that the system was composed of a heating element powered by an adjustable direct-current source, two copper blocks, a heat sink, and an infrared thermal imager (T420, FLIR Co., North Billerica, MA, USA). The copper block above the TIM was attached to the heating element with thermal grease and employed as the hot end. Meanwhile, the copper block below the TIM was adhered to the heat sink with thermal grease as well, and used as the cold end. The lateral surfaces of the copper blocks and the TIM were coated with carbon black to get uniform emissivity. When the heating element is working, the temperature of the copper blocks and the temperature gradient across the TIM can be recorded by the infrared thermal imager.

**Figure 8:**
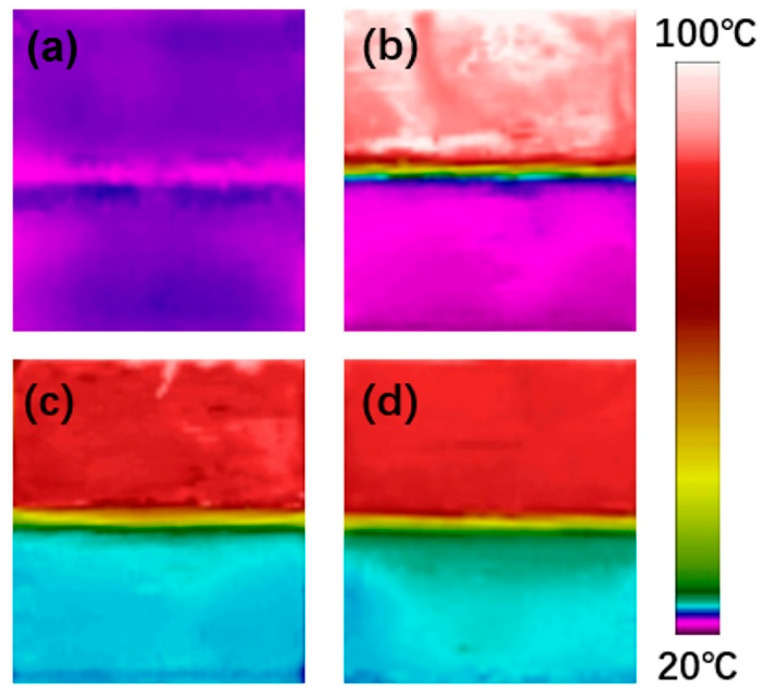
Temperature distributions before heating (**a**), using commercial polyurethane PU without phase change properties, SPU (**b**) and the rGOF/SPU composite as TIMs before (**c**) and after (**d**) heating.

Commercial PU without phase change properties, SPU, and the rGO/SPU composite were investigated by this in situ test system. The thickness of the TIM was 1.15 mm, while the areas of the heating element, copper blocks, and TIM were 2 × 2 cm. The performance of the TIMs was compared by the temperature differences of the two copper blocks: the more efficient the TIM was in heat dissipation, the lower the temperature difference between the hot end and the cold end. When the power supplied to the heating element was ~7.2 W, the distributions of temperature between the two copper blocks were in a steady state (Figure 8b–d). Although the thermal conductivity of the SPU was almost equal to that of a commercial PU, the temperature difference of the SPU TIM (64.3 °C) was 10.9 °C lower than that of commercial PU (75.4 °C). This is attributed to the final temperature (above 80 °C) being higher than the phase change temperature, and that the SPU transforms to an amorphous solid phase and lead to good interfacial wettability, causing increased contact area and reduced contact resistance. The rGOF/SPU composite achieved the best heat transfer performance with a temperature difference of 61.6 °C due to the higher thermal conductivity. Furthermore, no leakage was observed during the testing process as the hard segments restricted the movement of the soft segment molecular chains [29], indicating that the rGOF/SPU composite is an effective and stable TIM.

## 4. Conclusions

The rGO-coated PU foam-filled SPU composite was successfully prepared as a TIM with solid–solid phase change properties. It exhibited the advantages of proper phase change temperature, high thermal energy storage capacity, good wetting ability, and no leakage. For TIMs of PU and SPU with almost the same thermal conductivities, the solid–solid phase change properties were proved to significantly improve the heat transfer performance by increasing interfacial wettability. Adding rGOF further improved the thermal conduction of the composite with an enhancement of thermal conductivity by 63% at an ultra-low content of 0.8 wt.%. This work has provided a method to prepare high-performance thermal interface materials with solid–solid phase change properties and rapid heat transfer ability. The significant improvement of thermal conductivity and interfacial wettability has shown great potential for thermal management in electronic industries.
